# Targeting the Bacterial Orisome in the Search for New Antibiotics

**DOI:** 10.3389/fmicb.2017.02352

**Published:** 2017-11-27

**Authors:** Julia E. Grimwade, Alan C. Leonard

**Affiliations:** Department of Biological Sciences, Florida Institute of Technology, Melbourne, FL, United States

**Keywords:** antibiotic discovery, orisome, *oriC*, DnaA, initiation of bacterial DNA replication

## Abstract

There is an urgent need for new antibiotics to combat drug resistant bacteria. Existing antibiotics act on only a small number of proteins and pathways in bacterial cells, and it seems logical that expansion of the target set could lead to development of novel antimicrobial agents. One essential process, not yet exploited for antibiotic discovery, is the initiation stage of chromosome replication, mediated by the bacterial orisome. In all bacteria, orisomes assemble when the initiator protein, DnaA, as well as accessory proteins, bind to a DNA scaffold called the origin of replication (*oriC*). Orisomes perform the essential tasks of unwinding *oriC* and loading the replicative helicase, and orisome assembly is tightly regulated in the cell cycle to ensure chromosome replication begins only once. Only a few bacterial orisomes have been fully characterized, and while this lack of information complicates identification of all features that could be targeted, examination of assembly stages and orisome regulatory mechanisms may provide direction for some effective inhibitory strategies. In this perspective, we review current knowledge about orisome assembly and regulation, and identify potential targets that, when inhibited pharmacologically, would prevent bacterial chromosome replication.

## Introduction

The increase in life-threatening infections caused by multi-drug resistant bacteria has caused an urgent need for new antibiotics. Prevalence of drug-resistant bacteria can be partly attributed to over-use of antibiotics, both clinically and agriculturally (Ventola, 2015), but antibiotic resistance is an ancient phenomenon (D’Costa et al., 2011), and selection of resistant organisms is a predictable and inevitable consequence of antibiotic use. Complicating the problem is lack of diversity in current antibiotic targets; of the approximately 200 essential genes identified in bacteria, only a handful are currently targeted (Lewis, 2013). Because recent drug discovery efforts have focused largely on modifying existing scaffolds, any new drug that acts on molecular targets in the few exploited processes risks encountering pre-selected, resistance-causing mutations (Barker, 1999). Therefore, one logical way to combat antibiotic resistance is to expand the set of targeted essential processes and proteins. One unexploited process is assembly of the orisome, the nucleoprotein complex that mediates initiation of bacterial chromosome replication, a critical event in the bacterial cell cycle (Leonard and Grimwade, 2015). In this perspective, we review orisome assembly, and address whether or not orisomes contain molecular targets that are not only novel, but which might also lead to the development of clinically useful antibiotics.

## Orisome Assembly

All bacteria must duplicate their genomes before they divide into two identical daughter cells. With a few exceptions, all bacteria share fundamental molecular machinery responsible for triggering new rounds of DNA synthesis, comprising a unique chromosomal replication origin, *oriC*, and the conserved initiator protein, DnaA, a member of the AAA+ family of ATPases. The nucleoprotein complex formed by these two components is termed the orisome, which, when fully assembled, unwinds *oriC* DNA, and recruits replicative helicase, preparing the origin for the two replisomes required to bi-directionally replicate the circular genome (Wolański et al., 2014; Leonard and Grimwade, 2015).

**FIGURE 1:**
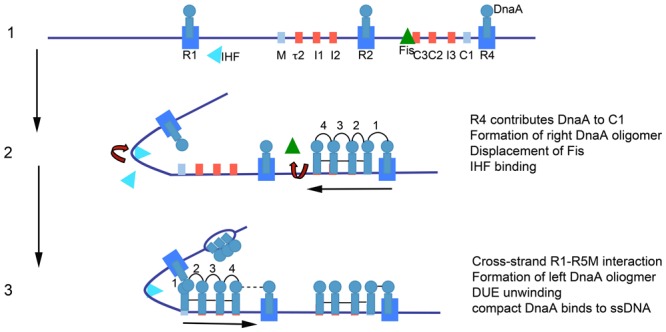
Model of staged orisome assembly. Stage 1: Immediately after initiation of chromosome replication, DnaA rebinds to high affinity R1, R2 and R4 sites. Fis is also bound, but IHF is not. Low affinity sites are unoccupied. Stage 2: DnaA bound to R4 recruits DnaA for binding to its proximal site, and DnaA then progressively fills the remaining arrayed sites. DnaA displaces Fis, and this allows IHF to bind to its cognate site. Stage 3: The bend induced by IHF binding allows DnaA, recruited by R1, to bind to R5M, and form a cross-strand DnaA interaction. DnaA then progressively fills the sites between R5M and R2. Coincident with completion of this stage, *oriC* DNA is unwound in the DUE, and DnaA binds to the ssDNA (Figure from Leonard and Grimwade, 2015).

The model for orisome assembly (**Figure 1**) is based largely on studies using *Escherichia coli* (Leonard and Grimwade, 2011, 2015). The orisome assembles from a persistent scaffold comprising three molecules of DnaA, interacting with three high affinity recognition boxes (R1, R2, and R4) (Cassler et al., 1995). The scaffold (stage 1) establishes a conformation of *oriC* that prevents premature unwinding and allows negative regulation by the DNA-bending protein Fis (Kaur et al., 2014). This scaffold also recruits and positions additional DnaA molecules for the next assembly stage (stage 2) (Miller et al., 2009). In stage 2, the N-terminal domain of DnaA bound to the high affinity R1 or R4 sites recruits DnaA to the proximal low affinity site (R5M or C1), followed by progressive binding of DnaA to the remaining lower affinity (non-consensus) binding sites; these sites preferentially bind DnaA-ATP (McGarry et al., 2004; Rozgaja et al., 2011). In the left region of *oriC*, DNA bending, assisted by the IHF protein, brings R1 and R5M into proximity to facilitate the cooperative DnaA site filling in *oriC*’s left half (Grimwade et al., 2000). Occupation of low affinity sites is required for the final stage (stage 3), when AT-rich DNA in a DNA Unwinding Element (DUE) is unwound, and DnaA-ATP associates with the single-stranded region (Yung and Kornberg, 1989; Speck and Messer, 2001), either in the form of a compact filament, or through interactions between ssDNA and domain III of DnaA bound to the left array of sites (Duderstadt et al., 2010; Ozaki and Katayama, 2012). DnaA in the DUE then recruits the replicative helicase and the helicase loader (DnaB and DnaC, respectively, in *E. coli*) (Sutton et al., 1998; Mott et al., 2008).

The instructions for orisome assembly are carried in all bacterial *oriC*’s in the form of precisely positioned recognition sites that direct DnaA binding (Rozgaja et al., 2011). DnaA is highly conserved and the consensus DnaA recognition motif in *E. coli* (5′-TTATCCACA) is also utilized by most bacteria (Schaper and Messer, 1995; Speck et al., 1997). However, there can be significant differences in the affinity each DnaA has for recognition sequences, particularly those that diverge from consensus (Zawilak-Pawlik et al., 2005; Ozaki et al., 2006). In addition, a database (DoriC^1^) (Gao et al., 2013) of over 1000 bacterial replication origins reveals a surprising variation in the arrangement, orientation and number of consensus or near consensus DnaA recognition sites among the *oriC*s of different bacterial types. Thus, although all orisomes contain a conserved protein (DnaA) and all perform the same essential function of origin activation, there is little obvious similarity in the set of instructions used to assemble them. How this diversity influences individual assembly stages and the transitions between those stages is not yet clear, and this lack of information could hamper identification of some conserved features essential for the mechanical aspects of origin activation that could be used as targets in antibiotic screens. Studies on orisomes outside of *E*. *coli* are ongoing, and the reader is referred to recent reviews discussing orisome assembly in different bacterial types (Wolański et al., 2014), as well as a review that includes strategies for rapid comparative analyses of diverse orisomes (Leonard and Grimwade, 2015).

## Orisome Regulatory Mechanisms: A Potential Guide to Effective Drug Targets?

Because more research is required before there is a unified paradigm for how orisomes trigger initiation, the best current strategy for identifying orisome targets may be to examine molecular mechanisms that regulate assembly. Logically, conserved mechanisms that inhibit orisome assembly will prevent initiation, and should provide “proof of principle” to justify targets as appropriate for pharmacological inhibition.

All orisomes are tightly regulated so that they trigger initiation of chromosome replication once, only once, and at the correct time in the cell cycle (Skarstad and Katayama, 2013). Delayed, or under-initiation leads to eventual chromosome loss, while re-initiation from the same origin can result in replication fork collapse and genome instability (Simmons et al., 2004). Like orisome assembly, regulation is best understood in *E. coli*, where two non-competing mechanisms, regulation of DnaA/*oriC* interactions, and regulation of cellular DnaA-ATP levels, predominate. Below, we review these two mechanisms and evaluate their possible utility as drug targets.

### Orisome Regulation by Controlling DnaA-*oriC* Interactions

In *E. coli*, DnaA binding to *oriC* is controlled both before and immediately after initiation by mechanisms that prevent completion of orisome assembly stages 2 and 3 (Leonard and Grimwade, 2005). Before initiation, the DNA bending protein Fis helps maintain the origin in a conformation that reduces DnaA’s ability to bind low affinity sites, until levels of DnaA increase enough to displace Fis from its recognition site (Ryan et al., 2004; Kaur et al., 2014). Since *E. coli oriC* contains multiple low affinity DnaA binding sites that preferentially bind DnaA-ATP (McGarry et al., 2004; Kawakami et al., 2005), orisome assembly cannot be completed until DnaA-ATP levels rise to a critical level. (Regulation of DnaA-ATP levels is discussed below.) After initiation, the SeqA protein binds hemimethylated GATC motifs in *oriC*, several of which are inside or overlap low affinity DnaA recognition sites (Lu et al., 1994; Skarstad et al., 2001). SeqA blocks DnaA-ATP from re-occupying low affinity sites and the DUE region for approximately one third of the cell cycle (Nievera et al., 2006).

It isn’t known how many bacterial origins contain low affinity recognition sites with preference for DnaA-ATP, and not all bacteria use Fis or SeqA to regulate orisome assembly (Brézellec et al., 2006; Madiraju et al., 2006). Regardless, the basic paradigm of controlling DnaA’s access to *oriC* as a way of regulating orisome assembly can be found in many bacterial types. For example, response regulators CtrA, MtrA, and HP1021 inhibit DnaA occupation of *oriC* in *Caulobacter crescentus, Mycobacteria tuberculosis*, and *Helicobacter pylori*, respectively, and by doing so, help prevent untimely initiations (Taylor et al., 2011; Donczew et al., 2015; Purushotham et al., 2015). *H. pylori* also uses DNA topology to regulate DnaA/*oriC* interactions (Donczew et al., 2014). In *Bacillus subtilis*, several proteins have been identified that negatively regulate initiation by inhibiting cooperative binding of DnaA at *oriC*; these include YabA (Merrikh and Grossman, 2011; Scholefield and Murray, 2013), DnaD (Bonilla and Grossman, 2012; Scholefield and Murray, 2013), and Soj (Scholefield et al., 2012). In several systems, orisome assembly is also controlled by positive regulators that increase DnaA binding to low affinity sites. In *E. coli* and *Caulobacter crescentus*, low affinity DnaA binding is stimulated by the DNA bending protein IHF (Grimwade et al., 2000; Siam et al., 2003). Additionally, the *E. coli* DiaA protein (Ishida et al., 2004), and its homolog in *H. pylori*, HobA (Natrajan et al., 2007; Zawilak-Pawlik et al., 2007, 2011), bind to DnaA’s domain I and increase weak site occupation.

The studies described above suggest that several different regions of DnaA could be targeted to inhibit DnaA binding. Obviously, blocking the DNA binding domain (domain IV) should inhibit all stages of orisome formation. Although protein–DNA interactions have not traditionally been considered to be “druggable” targets, recent studies have reported success in identifying inhibitors of DNA binding (Huang et al., 2016; Grimley et al., 2017). Further, inhibition of the self-oligomerization regions of DnaA in domains I and III should prevent cooperative binding and thus assembly stages 2 and 3 (Kawakami et al., 2005; Miller et al., 2009; Duderstadt et al., 2010; Scholefield and Murray, 2013). Like protein–DNA interactions, protein–protein interactions have not traditionally been favored as drug targets, but recent reports raise optimism that targeting DnaA oligomerization could be successful (Marceau et al., 2013; Voter et al., 2017).

Several other must be resolved before inhibition of DnaA’s access to *oriC* can be determined to be a practical antimicrobial strategy. First, it is not yet clear how much binding must be prevented to inhibit replication. All origins contain multiple DnaA binding sites (Leonard and Mechali, 2013), and studies that removed or inactivated DnaA recognition sites in *E. coli* chromosomal *oriC* revealed a tremendous plasticity in orisome assembly. Remarkably, deletion of the entire right region of *oriC* is tolerated in slow growing cells (Stepankiw et al., 2009). Additionally, directed mutations that knocked out binding to individual chromosomal *oriC* sites had little effect on viability (Weigel et al., 2001; Riber et al., 2009; Kaur et al., 2014). However, eliminating binding to more than one high affinity site did cause loss of viability (Kaur et al., 2014). Similar plasticity was noted in SeqA regulation of the number of occupied DnaA sites; even though loss of SeqA binding would be expected to allow DnaA re-binding at some *oriC* sites after initiation, mutating individual GATCs had little effect on initiation synchrony (Jha and Chattoraj, 2016). In *Bacillus*, some individual chromosomal *oriC* DnaA binding sites were shown to be essential, but others were not (Richardson et al., 2016). These studies, although by no means comprehensive, suggest that any pharmacological strategy should aim to inhibit DnaA binding at a majority of *oriC* sites, at least until future orisome studies reveal which sites are needed to assemble sub-complexes that carry out the essential mechanical functions of origin activation. Additionally, several studies suggest that assays used to screen for inhibitors of DnaA binding should be based on inhibiting chromosomal *oriC* rather than cloned origins, since inactivating individual sites is much more detrimental to plasmid *oriC* function (Weigel et al., 2001). Also, given the diversity in bacterial origin configurations (Leonard and Mechali, 2013), screens using a single bacterial type might not be sufficient to identify agents that act against a broad spectrum of bacteria. It might be necessary to utilize multiple types of bacteria, unless methodology is developed that allows the function of any chromosomal origin to be examined in an easily cultured strain. One strategy, involving heterologous origin transplantation, was described in a recent review (Leonard and Grimwade, 2015).

### Orisome Regulation by Controlling DnaA-ATP Levels

Based on seminal studies of *in vitro E. coli* DNA replication by the Kornberg lab (Sekimizu et al., 1987), DnaA-ATP is the active initiator form, and it is widely accepted that all bacteria share the requirement for DnaA-ATP in origin activation. In *E. coli*, DnaA-ATP levels are tightly regulated during the cell cycle to ensure precise initiation timing. Prior to the initiation step, DnaA-ATP levels rise due to new synthesis and a combination of recharging systems that include the DARS loci and acidic phospholipids in the membrane, reviewed in Skarstad and Katayama (2013). After initiation, the synthesis of DnaA-ATP is repressed for 1/3 of the cell cycle by SeqA, which binds to hemi-methylated GATC motifs in the *dnaA* promoter (Campbell and Kleckner, 1990). To inactivate DnaA-ATP, DnaA’s intrinsic ATPase activity is stimulated by the Hda protein associated with the β-clamp (DnaN) (Su’etsugu et al., 2004; Kim et al., 2017). Excess DnaA-ATP can also bind to a high capacity locus, termed *datA* (Kitagawa et al., 1998), which also stimulates DnaA-ATP hydrolysis (Kasho and Katayama, 2013).

The critical importance of mechanisms regulating DnaA-ATP levels in *E. coli* is demonstrated by the lethality observed in mutants, such as *dnaA*(cos) and *hda* null, that have lost the ability to inactivate DnaA-ATP by hydrolysis (Nishida et al., 2002; Felczak and Kaguni, 2009). DnaA(cos) carries two amino acid substitutions, one that prevents nucleotide binding (A184V), and another that stabilizes the mutated form (Y271H) (Simmons and Kaguni, 2003). Cells harboring *dnaA*(cos) are non-viable at 30°C, most likely due to over-initiation that results in co-directional replication fork collisions at stalled forks, leading to catastrophic double-stranded breaks (Simmons et al., 2004). A similar lethal phenotype is seen when Hda is inactivated, unless suppressor mutations arise (Riber et al., 2009; Charbon et al., 2011). Interestingly, although diverse suppressor mutations have been identified (Charbon et al., 2011), they all seem to cause tolerance of over-initiation by decreasing the chance of fork collisions, either by reducing initiation frequencies, or by preventing DNA lesions, such as oxidative DNA damage, that would slow forks (Charbon et al., 2014, 2017).

There are several aspects of DnaA inactivation mutants that are relevant to identifying antibiotic targets. First, lethality is caused by increasing, rather than decreasing the initiation frequency (Simmons et al., 2004). The run-away replication observed in DnaA(cos) mutants correlates with the inability to bind adenine nucleotide (Simmons et al., 2003), although it is not clear why loss of nucleotide binding leads to over-replication rather than orisome inactivation. Second, it is not yet known how many other bacterial types use regulation of DnaA-ATP levels as a regulatory mechanism. While some bacteria, such as *Caulobacter* and most enterobacteria, appear to have homologs of *hda* (Wargachuk and Marczynski, 2015), others, such as *Bacillus, Staphylococcus*, and *H. pylori*, do not (Katayama et al., 2010). DnaA in *B. subtilis* and *S. aureus* exchange bound ADP for ADP much more rapidly than *E. coli* does (Kurokawa et al., 2009; Bonilla and Grossman, 2012), and negative regulation of orisomes in these bacteria is focused mainly on DnaA-DNA interactions. Thus, screens to identify stimulators of DnaA hydrolysis may be ineffective in identifying broad-spectrum antimicrobials. In contrast, the growth inhibition/lethality caused when DnaA can’t hydrolyze ATP suggests that identification of inhibitors of ATP binding or ATPase activity, causing lethality by over-initiation, may be more fruitful. While targeting of the ATPase of AAA+ proteins is still in its infancy there are reports of successful inhibition of this protein class (Chou et al., 2011; Firestone et al., 2012). Targeting of DnaA’s ATPase, however, could generate suppressor mutations that reduce fork collisions (Charbon et al., 2017) and thus be prone to rapid resistance development. Possibly, this could be resolved by combination with an agent that inhibits DNA repair to counteract the actions of suppressor mutations (Simmons et al., 2004; Sutera and Lovett, 2006).

## Additional Considerations In Targeting Orisome Function

Obviously, any antibiotic acting on the orisome must enter the bacterial cell. This presents a problem with all bacteria, but particularly Gram negative bacteria, where the relatively impermeable outer membrane presents a potential barrier to drug delivery (Lewis, 2013; Brown, 2016). Until more is known about transport across the outer membrane, successful platforms to discover drugs affecting orisomes or any other intracellular target are likely to require living cells to augment or replace *in vitro* biochemical assays. While screen development is beyond the scope of this Perspective, we note that one cell-based assay, to identify agents that allow *dnaA*(cos) cells to grow at non-permissive temperature, has been described (Fossum et al., 2008), but failed to identify any small molecule inhibitors of DnaA function in a limited trial screen, although it is possible that lead compounds could be identified by screening a much larger library.

Of greater concern is generation of intra- or extra-genic suppressors, particularly if a new drug causes over-replication. Unfortunately, bacteria are adept in their ability to survive initiation perturbation. In cases where rapid development of resistance is expected, hybrid antibiotics or combination chemotherapy, where orisome inhibitors are combined with drugs that act on different pathways, should be considered. Alternatively, it might be useful to target features within DnaA that are shared by other proteins, since the majority of currently used successful antibiotics delay resistance development by attacking more than one target (Silver, 2011; Brown and Wright, 2016). One possible shared motif is the AAA+ domain, since the AAA+ domain of DnaC is quite similar to that of DnaA (Mott et al., 2008). Interestingly, hydrolysis of the ATP bound to DnaC is required before DnaB helicase can function (Mott et al., 2008), and it may be possible to identify inhibitors of DnaA’s intrinsic ATPase that also inhibit DnaB activation.

It is interesting that no natural product that inhibits orisome function has been identified in many years of antibiotic screening. This may be because the assays are not designed to identify drugs inhibiting this essential process, or that targeting the orisome is an inherently risky competition strategy for any bacteria, and so it rarely evolves. Regardless, the orisome appears to have potential as a novel and effective drug target, and its usefulness in antibiotic discovery should increase as more studies reveal conserved and non-conserved features of orisome assembly among bacterial types.

## Author Contributions

All authors listed have made a substantial, direct and intellectual contribution to the work, and approved it for publication.

## Conflict of Interest Statement

The authors declare that the research was conducted in the absence of any commercial or financial relationships that could be construed as a potential conflict of interest.

## Footnotes

1 http://tubic.tju.edu.cn/doric/index.php
